# The Role of HECT E3 Ubiquitin Ligases in Colorectal Cancer

**DOI:** 10.3390/biomedicines11020478

**Published:** 2023-02-07

**Authors:** Aiqin Sun, Yifei Chen, Xianyan Tian, Qiong Lin

**Affiliations:** School of Medicine, Jiangsu University, Zhenjiang 212013, China

**Keywords:** colorectal cancer, HECT E3 ligases, ubiquitination, tumor promoter, tumor suppressor, therapeutic strategy

## Abstract

Colorectal cancer (CRC) is estimated to rank as the second reason for cancer-related deaths, and the prognosis of CRC patients remains unsatisfactory. Numerous studies on gastrointestinal cell biology have shown that the E3 ligase-mediated ubiquitination exerts key functions in the pathogenesis of CRC. The homologous to E6-associated protein C-terminus (HECT) family E3 ligases are a major group of E3 enzymes, featured with the presence of a catalytic HECT domain, which participate in multiple cellular processes; thus, alterations in HECT E3 ligases in function or expression are closely related to the occurrence and development of many human malignancies, including—but not limited to—CRC. In this review, we summarize the potential role of HECT E3 ligases in colorectal carcinogenesis and the related underlying molecular mechanism to expand our understanding of their pathological functions. Exploiting specific inhibitors targeting HECT E3 ligases could be a potential therapeutic strategy for CRC therapy in the future.

## 1. Introduction

Despite extensive progress in early diagnostics, anticancer therapeutics, and improved clinical outcomes, colorectal cancer (CRC) remains the second leading cause of cancer mortality, with an estimated 935,000 deaths, representing 9.4% of all cancer deaths [1]. CRC presents as a heterogenous disease, and the exact mechanisms for CRC tumorigenesis and progression are complex and unclarified [2,3]. There are some diagnostic and prognostic biomarkers of CRC, such as mast cells (MCs), microRNAs (miRNAs), KRAS, and BRAF, identified in recent years [4]. However, there is not yet a clear recommendation about the clinical use of biomarkers in CRC. Uncovering the mechanisms of colorectal carcinogenesis and identifying novel molecular therapeutic targets for patients with CRC are still key issues.

Growing evidence emphasizes the importance of ubiquitination as a regulator in the development and progression of CRC. The ubiquitination process is highly conserved in all eukaryotes, which is important for controlling fundamental biological processes such as protein degradation, endocytosis, gene transcription, and autophagy [5,6]. Protein ubiquitination is a multistep reaction that can be achieved through a highly orchestrated enzymatic cascade [7]. Firstly, the ubiquitin activating enzymes (E1) are used to activate the ubiquitin molecule through forming a thioester bond between its Cys residue and the C-terminus of ubiquitin in an ATP-dependent manner. Next, the activated ubiquitin is loaded on the ubiquitin conjugating enzymes (E2). Finally, the ubiquitin ligases (E3) recruit E2-conjugated ubiquitin and mediate the transfer and attachment of ubiquitin to substrate proteins to form ubiquitinated substrates [8]. It was reported that only 2 E1s and about 40 E2s, but over 600 E3s, have been discovered in humans [9]. The ubiquitinated substrates have distinct fates and consequences, which are dictated by the type of ubiquitin modification produced on them [10]. Notably, K48- and K11-linked polyubiquitination direct substrates to proteasomal degradation [11]; while monoubiquitination and K63-linked polyubiquitination are related to nonproteolytic functions, such as subcellular localization, endocytosis, and autophagic degradation [11,12,13]. Along with other types of post-translational modifications, ubiquitination is a dynamic and reversible reaction; the removal of ubiquitin is carried out by deubiquitinating enzymes (DUBs) [14]. Approximately 100 DUBs have been identified in humans, which also play important roles in maintaining cellular function and homeostasis [14]. Within the ubiquitination process, E3 ligases largely determine specificity and versatility through the recognition and labeling of target substrates. To date, there has been a large class of proteins constituting the E3 ligases, which are defined as three major groups based on structural similitude [15]. The most abundant group is a really interesting new gene (RING) finger containing E3 ligases, which includes its derivatives, and function as scaffolds that facilitate ubiquitin’s direct transfer from E2 to the substrate [16]. The homologous to E6-associated protein C-terminus (HECT) domain containing E3 ligases are the second largest group, consisting of 28 members, which have intrinsic catalytic activity that form a covalent thioester intermediate that accepts ubiquitin from the E2 prior to its destined substrate [10]. In recent years, the RING-in-between-RING (RBR) E3 ligases have been identified as a third group of E3s, sharing common features with the former two E3s and employing a hybrid mechanism of RING and HECT E3s to conjugate ubiquitin to the specific substrate [17].

Recent studies show that an increasing number of E3 ligases attribute key roles in regulating colorectal carcinogenesis and progression, and the majority of these E3s act as either tumor promoters or tumor suppressors, while some others seem to play a dual role. HECT E3 ligases have been discovered to target signaling molecules in PI3K/AKT and WNT pathways to modulate their activation in CRC. Reviews regarding the function of E3 ligases in CRC are mostly focused on the RING family [18,19]. Here, we summarize the current evidence accumulated concerning the diverse roles of HECT family members in CRC.

## 2. The Classification of HECT E3 Ligases

In addition to invariably HECT domain for catalyzing the transfer of ubiquitin to substrates at their C-terminus, HECT E3 ligases recognize and recruit substrates through the N-terminal specific protein–protein interaction domains [7]. The HECT E3 ubiquitin ligases can be assembled into three subfamilies based upon the presence of distinct regions at the N-terminus: NEDD4 (also designated C2-WW-HECT), HERC, and ‘other’ HECT E3s [20].

**Figure 1:**
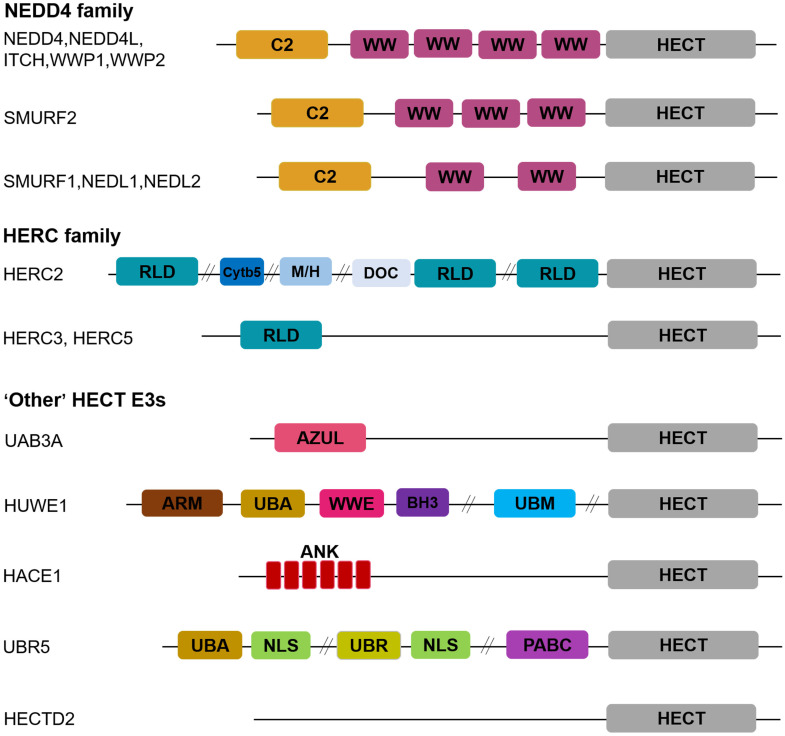
Structural features of the HECT E3 ligases. HECT E3 ligases are classified into three subfamilies: NEDD4, HERC, and ‘other’ HECT E3 family. The NEDD4 family is characterized by C2 and WW domains, the HERC family contains one or more RLD domains, and the ‘other’ HECT E3 family carries distinct structural domains at the N-terminus. The domain abbreviations used are as follows: C2, Calcium-dependent lipid binding domain; WW, WW domain; HECT, homologous to E6AP C-terminus; RLD, regulator of Chromosome Condensation 1 repeat-like domain; Cytb5, cytochrome b5-like heme/steroid-binding domain; M/H, MIB-HERC2 domain; DOC, APC10/DOC domain; AZUL, amino-terminal Zn-binding domain of UBE3A ligase; ARM, armadillo repeat-containing domain; UBA, ubiquitin-associated domain; WWE, WWE domain; BH3, Bcl-2 homology 3 domain; UBM, ubiquitin-binding motif; ANK, Ankyrin repeat-containing domain; NLS, nuclear localization sequence; UBR, ubiquitin-recognin box; PABC//MLLE, polyadenylate-binding protein C-terminal domain.

As the best-characterized and most studied group of HECT E3s, the NEDD4 subfamily has nine mammalian members in humans whose structures contain an N-terminal C2 domain and multiple WW domains preceding the HECT domain [21] (Figure 1). The C2 domain is a Ca2+-dependent binding motif mainly for cell membrane substrate targeting [22], whereas the WW domains recognize proline-rich peptide motifs and phosphorylated serine/threonine-proline sites in substrates or accessory proteins [23].

HERC subfamily comprises six members in humans, HERC1–HERC6, containing one or more chromosome condensation 1 (RCC1)-like domains (RLDs) at their N-terminus for substrate binding and recruitment [24] (Figure 1). HERC1 and HERC2 belong to large HERC E3s, carrying more than one RLD and additional domains, which are the largest HECT ligases with exceeding 500 kDa molecular weights, whereas HERC3-6 constitutes the small HERC E3s with molecular weights of approximately 120 kDa that possess only one RLD domain [25].

The remaining 13 HECT E3s lack WW or RLD domains and carry distinct structural domains at the N-terminus, named ‘other’ HECT ligases (Figure 1). Multiple reports have shown that the aberrant expression, mutations, and deregulated activity of HECT E3 ligases disrupt normal cellular processes and signaling pathways and induce the occurrence and development of many human malignancies [7,26,27]. This review focuses on the potential role and related molecular mechanisms of HECT E3 ligases in CRC carcinogenesis and progression.

More and more studies support the view that HECT E3 ligases play crucial roles in various human pathologies, from neurodegenerative disorders to cancers [7]. Abnormal expression, mutations, or deregulated activity of HECT E3 ligases have been discovered in different cancers. HECT E3 ligases can promote or prevent cancer, mainly depending on the ubiquitination of substrates. For example, PTEN, recognized as a tumor suppressor, has been revealed to be regulated by members of NEDD4 subfamily of HECT E3 ligases singly or cooperatively [28]. Indeed, multiple studies have indicated prominent roles of HECT E3 ligases in CRC tumorigenesis. The aim of this review is to discuss the emerging role of HECT E3s as diagnostic and prognostic biomarkers for CRC, revealing new avenues for the prevention and treatment of CRC based on targeting E3 ligases.

## 3. The Involvement of HECT E3 Ligases in Colorectal Cancer

### 3.1. NEDD4 Family

#### 3.1.1. NEDD4

NEDD4, also referred to as NEDD4-1, has been reported as one of the central drivers in CRC. NEDD4 is abundantly expressed in CRC, and no significant association between the NEDD4 expression and PTEN protein levels has been observed [29,30]. Further study demonstrated that NEDD4 is involved in the ubiquitination of FOXA1, which transcriptionally activates miR-340-5p to inhibit ATF1 expression and consequently diminishes the promotion of NEDD4 on CRC progression [31]. These data suggest that NEDD4 might function as an oncogene in CRC. N-myc downstream-regulated gene 1 (NDRG1) was significantly downregulated in several cancers. Notably, NDRG1 could antagonize NEDD4-mediated ubiquitination degradation of p21 to serve as a tumor suppressor in CRC [32]. However, there is another line evidence supporting a tumor-suppressive role of NEDD4. A loss of heterozygosity or mutations of NEDD4 was found in CRC [33,34]. In vivo experiment has found that knockout of NEDD4 augments colorectal tumor growth in APC+/min mice [35]. Specifically, the loss of NEDD4 elevated the expression level of LEF1 and YY1 in APC mutant-driven CRC, thereby promoting the activation of the WNT signaling pathway [35]. In addition, NEDD4 mediates LGR4/5 and DVL2 lysosomal and proteasomal degradation, resulting in inactivation of the WNT/β-catenin signaling pathway and inhibition of the susceptibility and progression of colorectal tumors [36].

#### 3.1.2. NEDD4L

NEDD4L, also known as NEDD4-2, shows high homology with NEDD4. Accumulating evidence has highlighted a role for NEDD4L as a tumor suppressor in CRC (Table 1). Compared with normal tissues, NEDD4L expression in CRC tissues is decreased significantly, and the expression of NEDD4L results in inhibition of the WNT/β-catenin signaling pathway, without affecting tumor growth [37]. In another study, NEDD4L targets the RSPO receptor LGR4/5 and DVL2 for degradation to inactivate the WNT/β-catenin signaling pathway and attenuate intestinal stem cell priming, leading to tumor predisposition and progression inhibition [36]. Furthermore, STK35 was identified as a new downstream substrate of NEDD4L, thereby inhibiting the AKT signaling pathway, and ultimately exerting anti-CRC functions [38].

#### 3.1.3. ITCH

ITCH is implicated in multiple important pathways and acts on tumorigenesis [89]. Huang et al. reported that Cir-ITCH and ITCH could repress CRC tumorigenesis by inhibiting the WNT/β-catenin pathway [39]. Furthermore, it was identified in CRC that ROR-γt, a lineage marker for the Th17 cell population, is the downstream target of ITCH, which exerts its anti-colon-carcinogenic role by attenuating IL-17 expression [40]. Consistently, another study showed that ITCH-mediated ROR-γt degradation is impeded by CRNDE-h through the disruption of ITCH/ROR-γt interaction, thus promoting Th17 cell differentiation in CRC [41]. In addition, CCDC68 can upregulate ITCH expression to promote proteasome degradation CDK4, leading to the inhibition of CRC cell growth [42]. Moreover, Park et al. demonstrated that ITCH interacting and ubiquitinating FLIP for degradation may be induced by treatment with Codium F2, which sensitizes CRC cells to TRAIL-induced apoptosis [43]. These studies mainly support ITCH as a tumor suppressor in CRC (Table 1).

#### 3.1.4. WWP1

WWP1 is a multifunctional protein and has been involved in numerous human tumors [90]. Upregulated WWP1 has been reported in CRC and is associated with tumor size, T classification, distant metastasis, TNM stage, and adverse patient prognosis [44]. WWP1 depletion attenuates the proliferative and invasive phenotype of CRC cells, likely by inactivating the PTEN/AKT signaling pathway [44]. In addition, the negative correlation between WWP1 and miR-16 was found in CRC, and miR-16 could target WWP1 to suppress CRC tumor growth [45]. WWP1 seems to exert pro-oncogenic functions in CRC (Table 1), and future studies are deserved to verify the role of WWP1 in CRC.

#### 3.1.5. WWP2

Like WWP1, WWP2 is also involved in tumor progression through regulating the PTEN/AKT pathway in CRC. It is known that WWP2 can directly bind to and degrade the tumor suppressor PTEN and promote the PI3K/AKT pathway [46]. Wang et al. found that Linc02023 could specifically interact with PTEN to prevent the WWP2-mediated ubiquitination degradation of PTEN, which in turn inhibits the PI3K/AKT pathway, thus suppressing the tumorigenesis of CRC (Table 1) [47].

#### 3.1.6. SMURF1

In CRC, the elevated expression of SMURF1 was observed, and resulted in robust cancer progression and poor prognosis [48,49]. The mechanism included the activation of its ubiquitin ligase activity and enhanced pre-rRNA synthesis by its mediated RRP9 neddylation. Moreover, aberrantly expressed SMURF1 in colon cancer samples inversely correlated with the expression of CKIP-1, which repressed the SMURF1 synthesis and promoted SMURF1 autoubiquitination and degradation, thereby suppressing colon cancer cell growth and migration [50]. Recent studies found that decreasing SMURF1 enhances the chemosensitivity of CRC to 5-FU, gemcitabine, and cisplatin treatment [51,52]. In addition, a host of studies have found that SMURF1 itself is largely affected by numerous upstream factors. For instance, SMURF1 was phosphorylated at threonine residues by IRAK2, motivating its autoubiquitination and degradation, leading to an altered cascade of ER effectors to repress cell growth and induce apoptosis of CRC cells [53]. Moreover, SMURF1 can be targeted by MicroRNA-125a for CRC prevention and treatment [54]. In addition, SMURF1 was reported to be transcriptionally activated by FOSL1, which aggravates SMURF1-induced ubiquitination and degradation of FBXL2, resulting in the induction of WNT/β-catenin signaling to potentiate EMT, proliferation, and metastasis of CRC [55]. Thus, SMURF1 inhibition might act as an antitumor therapeutic option in CRC.

#### 3.1.7. SMURF2

The upregulation of SMURF2 predicted a favorable prognosis of patients with CRC and liver metastases, and the tumor-suppressive role of SMURF2 was found to be associated with cell migration and EpCAM expression [56]. In addition, SMURF2 was reported to restrain CRC promotion via targeting SATB1, ChREBP, SIRT1, FUBP1, YY1, and RhoA as its major substrates for ubiquitination degradation [57,58,59,60,61,62]. Moreover, Pu’s group found that SMURF2 protein expression was induced by schisandrin B and that SMURF2 expression is negatively correlated with SIRT1 expression, thus hindering cell growth and metastasis of colon cancer [63]. In contrast to the above studies, it has been reported that SMURF2, together with UBCH5, stabilize the oncoprotein KRAS [64]. Specifically, SMURF2 loss reduces colony survival in colon carcinoma induced by mutant KRAS in nude mice [64]. Furthermore, Klupp et al. identified that SMURF2 expression was elevated in CRC specimens and that the elevation of SMURF2 was significantly associated with impaired overall survival in microsatellite stable CRC, but not in microsatellite instable CRC [65], but the specific mechanism is not quite clear.

#### 3.1.8. NEDL1

NEDD4-like ubiquitin protein ligase-1 (NEDL1), also known as HECW1, consists of an N-terminal C2 domain, two WW domains, and a C-terminal catalytic HECT domain. It is reported to play an important role in neurological disease through the ubiquitination and degradation of mutant SOD1 and Dvl1 [91]. Moreover, NEDL1 can directly interact with C-terminus of p53 and increase the transcriptional activity of p53 in a manner that is less dependent on E3 activity, and subsequently promotes p53-mediated apoptotic cell death [92]. However, the extent of NEDL1 contribution to colorectal carcinogenesis is still far from being understood. The only published study reported that NEDL1 interacts with RNF43, a RING E3 ubiquitin ligase, which is overexpressed in CRC and binds to p53, leading to the suppression of p53-mediated transcriptional activity [93].

#### 3.1.9. NEDL2

NEDL2 (also known as HECW2) shows large homology with NEDL1, playing a role in regulating mitosis, enteric nervous system, and kidney development. To date, the function of NEDL2 in CRC remains poorly understood. Lu et al. found that the protein and mRNA levels of NEDL2 were significantly elevated in colon tumor tissues [94]. In addition, NEDL2 was identified as a novel substrate of APC/C-Cdh1 during mitotic exit and regulated the metaphase-to-anaphase transition [94]. However, the relationship between the imbalance of NEDL2 in CRC tumorigenesis and mitotic progression is not clear yet, and further study is needed.

### 3.2. HERC Family

#### 3.2.1. HERC2

HERC2 is a giant protein featured with multiple conserved regions that has been linked to different types of cancer. At present, little is known about the role of HERC2 in CRC. Yoo et al. showed that frameshift mutations of HECR2 were observed in GC and CRC with microsatellite instability [66]. HERC2 has been reported to regulate p53 oligomerization and the MDM2–p53 pathway [67,68]. A recent study speculated that HERC2-mediated p53 ubiquitination and degradation was inhibited by BTF3, which functions as an oncogene in CRC (Table 1) [69], but functional analysis of HERC2 and p53 with BTF3 needs subsequent study.

#### 3.2.2. HERC3

The same as HERC2, frameshift mutations of the HERC3 gene have also been identified in both GC and CRC with microsatellite instability [66]. Until recently, a research team focused on the role and mechanistic studies of HERC3 in CRC. They discovered that the expression of HERC3 was significantly downregulated in CRC tumor tissues and cell lines, and the downregulated HERC3 was closely related to shorter overall survival and disease-free survival in CRC patients [70]. The ectopic overexpression of HERC3 can inhibit the migration, invasion, and metastasis of CRC, while knockdown of HERC3 promoted migration, invasion, and metastasis [70]. Further mechanism studies have shown that HERC3 regulates the TGF-β/Smad2/3 signal through ubiquitination and proteasomal degradation EIF5A2, thereby inhibiting the epithelial–mesenchymal transition (EMT) of CRC [70]. In another study, the team showed that upregulation of HERC3 levels predicted lighter and smaller tumors of patients with CRC, decreased CRC cell growth, and arrested the cell cycle, probably via targeting RPL23A as its major substrate for degradation [71]. Collectively, these results indicate that HERC3 may be a potential tumor suppressor in CRC (Table 1).

#### 3.2.3. HERC5

HERC5 was originally discovered as an IFN-induced HECT-type E3 ligase, primarily regulating virus infection, immune response, and cell cycle. Several studies have established a link between HERC5 and in a wide range of cancers, including glioblastoma, breast cancer, lung cancer, and hepatocellular carcinoma [95]. Recently, Zhu et al. determined that the downregulation of HERC5 in human CRC attenuated its ubiquitination degradation of CtBP1, which inhibits apoptotic signaling by assembling into a transcriptional complex with HDAC1 and c-MYC, ultimately promoting the development of CRC [72]. At present, there is only one study on HERC5 in CRC has been conducted, and further exploration is needed.

### 3.3. ‘Other’ HECT E3 Family

#### 3.3.1. UBE3A

UBE3A, also known as E6AP, is a founding member of the HECT E3 ligases and is associated with HPV-related cervical carcinogenesis [96]. A study discovered that UBE3A may play a pivotal role in the development of colorectal cancer. NDRG2 was found to block UBE3A-mediated ubiquitination degradation of estrogen receptor beta (ERβ) by inhibiting the ability of UBE3A to combine with ERβ, thus playing a role in suppressing the proliferation of CRC cells [73]. Overall, UBE3A might act an oncoprotein in CRC (Table 1).

#### 3.3.2. HUWE1

Interestingly, in CRC, whether HUWE1 (also named Mule, HectH9, ARF-BP1, HSPC272, Ib772, URE-B1, E3 Histone, and LASU1) functions as a tumor promoter or a tumor suppressor remains controversial. Previous studies have shown that HUWE1 was overexpressed in CRC tumor tissues, and HUWE1 expression directly correlates with tumor progression [74,75]. Adhikary et al. also demonstrated that HUWE1 mediates K63-polyubiquitination of c-MYC, thereby promoting the stabilization of transcriptional activation of MYC and recruitment of coactivator p300, and ultimately stimulating CRC tumor cell proliferation [74]. Moreover, the investigation of Yoon et al. has shown that HUWE1 reduces the transcriptional activity of p53 through a ubiquitin-dependent degradation pathway, thus exerting oncogenesis effects [75]. Furthermore, small molecule inhibitors have been identified, which leads to the inhibition of HUWE1, thereby stabilizing MIZ1 and contributing to repressing MYC-activated target genes in CRC cells [76]. However, another study discovered that the deletion of HUWE1 in an APC-based mouse model of colorectal cancer accelerates tumorigenesis by increasing the levels of MYC and DNA damage accumulation [77]. In CRC, β-catenin stability was found to be negatively regulated by HUWE1, resulting in quenching the abnormal activation of the WNT signal [78]. Moreover, Park’s group identified that HUWE1 is required for proteasomal degradation of Mcl-1 to sensitize the effect of metformin on TRAIL-induced apoptosis of CRC cells [79]. Taken together, HUWE1 plays a dual role in CRC, and its function is reflected by the variety of substrates that HUWE1 regulates (Table 1).

#### 3.3.3. HACE1

Abnormal methylation and low expression of HACE1 are observed in CRC, as well as in GC, which are further proved to correlate with poor clinicopathological characteristics of patients [80,81]. Additionally, loss of HACE1 could predispose animals to colonic inflammation and carcinogenesis in vivo, and impairs TNF-driven NF-kB activation and apoptosis in vitro and in vivo [82]. Importantly, these are mainly due to the deregulation of the TNFR1-mediated RIP3 kinase signaling [82]. One latest report showed that overexpression of HACE1 reduced the protein levels of YAP1 to increase the activation of the Hippo pathway, thus inhibiting the proliferation, invasion, migration, and EMT of CRC cells [81]. However, the molecular mechanism of HACE1 in regulating the YAP1 pathway needs further investigation.

#### 3.3.4. UBR5

Gene mutations of UBR5 (also named EDD1, EDD, DD5, HYD, or KIAA0896) have been identified in CRC [97]. Interestingly, the role of UBR5 in CRC is controversial. One previous study indicated that UBR5 interacts with and upregulates tumor suppressor APC to inhibit the Wnt/β-catenin signaling pathway, indicating that UBR5 could act as a potential tumor suppressor in CRC [83], whereas more studies have suggested that UBR5 functions as an oncoprotein. UBR5 is highly expressed in CRC, and its high expression is negatively associated with prognosis [84,85]. Moreover, UBR5 has been shown to promote the growth of CRC cells and inhibit apoptosis [86]. The mechanism includes its mediated degradation of tumor suppressor esophageal cancer-related gene 4 (ECRG4) [85] and P21 [86], or nonproteolytic ubiquitination of β-catenin to activate transcription [87].

#### 3.3.5. HECTD2

Recently, another member of the ‘other’ HECT E3 family protein, HECTD2, containing no known N-terminal substrate binding domains, was also found to involved in CRC. Upon treatment with propionate, HECTD2 was upregulated and mediated the ubiquitination and proteasomal degradation of EHMT2, thus upregulating the expression of TNFAIP1, which is a direct target of EHMT2 for the induction of apoptosis of CRC cells [88]. The functions and related mechanism of HECTD2 in CRC need further exploration.

## 4. HECT E3 Ligases as Therapeutic Targets

**Figure 2:**
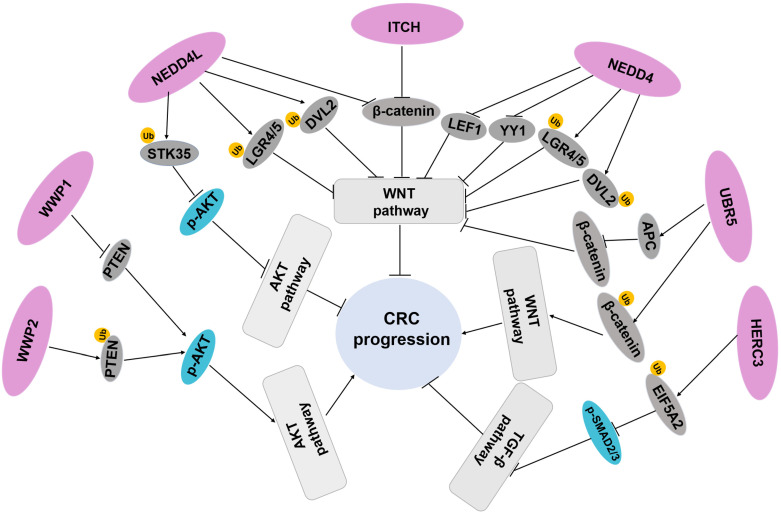
Regulation of important signaling pathways by HECT E3 ligases in CRC. NEDD4 and NEDD4L mediate RSPO receptor LGR5 and DVL2 lysosomal and proteasomal degradation, resulting in inactivation of WNT/β-catenin signaling pathway and inhibition of susceptibility and progression of colorectal tumors. NEDD4 also exerts tumor-suppressive functions by downregulating the protein level of LEF1 and YY1. NEDD4L causes ubiquitination and degradation of STK35 and block AKT pathway to inhibit CRC progression. ITCH represses CRC tumorigenesis by downregulating the protein level of β-catenin, and WWP1 drives CRC progression by activating AKT signaling pathway. WWP2 executes its regulation of the AKT signaling pathway through its E3 ligase activity. HERC3 promotes ubiquitination and degradation EIF5A2 to inactivate the TGF-β/Smad2/3 signaling in CRC development. UBR5 can mediate nonproteolytic ubiquitination of β-catenin to activate WNT pathway in CRC, and UBR5 also inhibits the Wnt/β-catenin pathway by upregulating APC.

Given the critical role exerted by the HECT E3 ligases in the regulation of diverse and important cellular pathways, including WNT, YAP-Hippo, TGF-β, and PI3K/AKT in CRC (Figure 2), these enzymes represent attractive therapeutic targets. Some molecules have been already developed to inhibit the HECT E3 ligase, such as the general small molecule heclin, which induces the oxidation of HECT domain catalytic cysteine to block several protein of HECT E3 ligases [98]. Clomipramine, an antidepressant drug, specifically inhibits the HECT catalytic activity of ITCH [99].

At present, the number of small molecules that selectively inhibit HECT E3 ligases and exert antitumor effects in CRC is very limited. BI8622 and BI8626, identified through high-throughput screening, were found to inhibit the enzyme activity of HUWE1, repress MYC-dependent transactivation, and restrict the growth of colorectal cancer cells [76]. However, it remains unclear how these two compounds modulate HUWE1. Due to the high conservation of the HECT domain and extremely complex functions of these enzymes, it is a major challenge to develop specific inhibitory compounds.

## 5. Conclusions and Future Perspectives

In conclusion, according to their specific contribution to CRC, HECT E3 ligases discussed here can be reassembled as tumor promoters, tumor suppressors, and bifunctional proteins (Table 1). The context-dependent roles of HECT E3 ligases in CRC are reflected by substrates and regulators of themselves. Additionally, the underlying mechanisms have remained remarkably elusive, although there are abundant studies proving that HECT E3 ligases exert a central role on CRC in both ubiquitination-dependent and ubiquitination-independent manners. In-depth mechanistic research should be carried out to expand our understanding of the function and role of HECT E3 ligases in CRC carcinogenesis and progression. We have reason to believe that the development and clinical application of HECT E3 ligase inhibitors for CRC treatment will be achieved in the near future.

## Figures and Tables

**Table 1:** HECT E3 ligases in human colorectal cancer.

Subfamily	E3	Expression	Substrates	Pathway	Role in CRC	Reference
NEDD4	NEDD4	Overexpression Loss of heterozygosity mutations	FOXA1 P21 LGR4/5 DVL2	WNT	Bifunctional protein	[29,30,31,32,33,34,35,36]
	NEDD4L	Decreased	LGR4/5 DVL2 STK35	WNTAKT	Tumor suppressor	[36,37,38]
	ITCH	Downregulation	ROR-γt CDK4 FLIP	WNT	Tumor suppressor	[39,40,41,42,43]
	WWP1	High expression	unknown	AKT	Tumor promoter	[44,45]
	WWP2	Upregulation	PTEN	AKT	Tumor promoter	[46,47]
	SMURF1	Upregulation	FBXL2	WNT	Tumor promoter	[48,49,50,51,52,53,54,55]
	SMURF2	Upregulation	SATB1 ChREBP SIRT1 FUBP1 YY1 RhoA	WNT	Bifunctional protein	[56,57,58,59,60,61,62,63,64,65]
HERC	HERC2	Frameshift mutations	P53	/	Tumor promoter	[66,67,68,69]
	HERC3	Frameshift mutations Downregulation	EIF5A2 RPL23A	TGF-β	Tumor suppressor	[66,70,71]
	HERC5	Downregulation	CtBP1	/	Tumor suppressor	[72]
‘Other’ HECT	UAB3A	/	ERβ	/	Tumor promoter	[73]
	HUWE1	Overexpression	c-MYC Mcl-1	WNT	Bifunctional protein	[74,75,76,77,78,79]
	HACE1	Downregulation Hypermethylation	TRAF2	NF-kBYAP-Hippo	Tumor suppressor	[80,81,82]
	UBR5	Overexpression Mutations	β-catenin ECRG4 P21	WNT	Bifunctional protein	[83,84,85,86,87]
	HECTD2	/	EHMT2	/	Tumor suppressor	[88]

## Data Availability

Not applicable.
